# A Medico-Legal Treatise on Malpractice, Medical Evidence and Insanity

**Published:** 1881-06

**Authors:** 


A Medico-Legal Treatise on Malpractice, Medical Evidence and Insanity, comprising the elements of Medical Jurisprudence. By Jno. J. Elwell, M.D., one of the editors of new edition of Bower's Law Directory, etc., etc. Fourth edition. Revised and enlarged. Baker, Voorhis & Co., New York. 1881.

This is a volume of five hundred and odd pages, gotten up in regular library binding, and large clear print. The medico-legal bearings of the book render it a desirable acquisition to the library of both the lawyer and physician. The general principles of law, defining the civil responsibilities and duties of physicians, are set forth with citations of illustrative cases.

